# Pathological Society of Philadelphia

**Published:** 1879-01

**Authors:** 


### PATHOLOGICAL SOCIETY OF PHILADELPHIA.

The President, Dr. Lenox Hodge, in the chair.

C. S., æt. 29, a sailor, died in my ward at the Episcopal Hospital, on October 12th, from catarrhal phthisis. Several months before coming under my care he had suffered with cystitis, for which disease he had been treated in the surgical ward of the hospital.

When admitted to the medical ward he presented all the ordinary rational symptoms and physical signs of advanced phthisis. His urine was examined, though there were no symptoms directing attention particularly to the kidneys or bladder, and found to be of low specific gravity, 1012, and to contain a small amount of albumen. The autopsy was made nine hours after death. On opening the thoracic cavity the lungs were found to be bound to the chest-walls by numerous pleuritic adhesion. The upper lobes of both lungs contained large cavities, while the remainder was infiltrated with caseous material. The heart was normal in size, its muscular tissue was pale and somewhat flaccid, and one leaflet of each of the semilunar valves was fenerated. The liver was large and fatty. The kidneys were quite different in appearance. The left was slightly enlarged, and presented the gross characteristics of the "large white kidney." The right was also larger than normal, and had a lobulated appearance externally; on section this lobulation was found to be due to saccular dilation of the pelvis and calyxes, the various sacs being filled with a turbid, urinous fluid, and having thick walls. The largest sac, nearly an inch in diameter, was situated in the lower part of the kidney, and approached very near the external surface, leaving only a thin layer of condensed leather-like renal tissue. The glandular structure of the rest of the kidney was not reduced in thickness, but was much firmer than normal, felt rough to the finger, and was studded with little nodules, which to the unaided eye appeared to be collections of miliary tubercles. In removing the kidney the ureter was torn off a short distance from the organ; the tube in this position was moderately dilated, and its wall was thickened and friable. The mucous membrane of the bladder was thickened and rough, and was covered with tenacious mucus; on the peritoneal surface a large number of small nodules, about as large as split peas, were observed. Similar nodules were also found on the intestinal peritoneum.

*Report of the Committee on Morbid Growths.*—"A microscopical examination of a section of the kidney presented by Dr. Starr shows the glandular part of the organ to be very much altered; the tubules are to a great extent deprived of their cells, and surrounded by fibrillar connective tissue in a state of proliferation. The capsules of the Malpighian bodies present a similar arrangement of structure. The miliary nodules scattered throughout the organ consist of a central granular mass, the periphery of lymphoid cells, in a stroma of delicate, reticulated fibrous tissue. The organ exhibits the lesion of acute tuberculosis.

"The new formations situated in the peritoneum covering the bladder are composed of elements similar to those found in the nodules of the kidney, and have a like arrangement.

*Gelatinous Arthritis of Knee.*—Presented by Dr. H. Lenox Hodge for Dr. John Ashhurst, Jr.—The specimen presented is the lower extremity of the femur, articulating surface of the tibia, and patella, from a patient whose thigh had been amputated the day before by Dr. John Ashhurst, Jr., at the Children's Hospital. The case was one of arthritis in a child of ten years, the subject of hereditary syphilis, who had been under treatment in the hospital for more than eighteen months. The first incision had been made as if for excision of the knee, but, the bone disease having been found too extensive to justify this operation, anterior and posterior flaps were cut, and the thigh amputated at its lower third. The specimen showed very well the characteristic gelatiniform change in the synovial and cartilaginous tissues, while the bone was markedly carious.

November 20—The flaps united by adhesion, and in ten days, the ligature having come away, the patient was looked upon as convalescent. Uninterrupted recovery followed the operation.

*Primary Cancer of the Intra-thoracic Glands.*—The patient, a blacksmith, æt. 40, was admitted to the Philadelphia Hospital suffering with large pleural effusion on left side, extreme dyspnœa, husky whispering voice, and slight dysphagia. The history was obscure. It appeared that for two years there had been occasional dysphagia and increasing huskiness of voice. For past three months there had been increasing dyspnœa. On careful examination, the physical signs of pressure on right bronchus were detected, and some hard and enlarged lymphatic glands were found in left supraclavicular space.

The diagnosis was made of primary cancer of intra-thoracic glands, pressing on right bronchus, œsophagus, trachea, and azygos vein. Death occured suddenly from syncope.

The autopsy revealed cancerous disease of the glands in both anterior and posterior mediastinum, forming masses of considerable size. The right bronchus was compressed. The œsophagus and descending thoracic aorta were partly imbedded in the cancerous growth. The azygos and hemiazygos veins were involved, and so imbedded that it was impossible to dissect them out. Large pleural effusion existed in the left side.

*Report of the Committee on Morbid Growths.*—"The specimen of intra-thoracic new formation presented by Dr. Pepper, and referred to the Committee on Morbid Growths, is found, by microscopic examination, to consist of a fibrous tissue stroma arranged so as to form alveolar spaces, in which spaces are seen epithelial cells. This arrangement of structure is characteristic of carcinoma—variety scirrhus. The enlarged glands from the neck have undergone a similar metamorphosis, as shown by microscopic examination.—*Medical Times.*

